# Coronary heart disease incidence and mortality, and all-cause mortality among diabetic and non-diabetic people according to their smoking behavior in Finland

**DOI:** 10.1186/s12971-017-0113-3

**Published:** 2017-02-02

**Authors:** Noël C. Barengo, Yvonne Teuschl, Vladislav Moltchanov, Tiina Laatikainen, Pekka Jousilahti, Jaakko Tuomilehto

**Affiliations:** 1 Department of Medical and Population Health Sciences Research, Herberth Wertheim College of Medicine, Florida International University, 11200 SW 8th Street, AHC2, Miami, FL 33199 USA; 2 Department of Neurosciences and Preventive Medicine, Danube-University Krems, Krems, Austria; 3 Department of Health, National Institute for Health and Welfare, Helsinki, Finland; 4 Institute of Public Health and Clinical Nutrition, University of Eastern Finland, Kuopio, Finland; 5 Hospital District of North Karelia, Joensuu, Finland; 6 Dasman Diabetes Institute, Dasman, Kuwait; 7 Diabetes Research Group, King Abdulaziz University, Jeddah, Saudi Arabia

**Keywords:** Smoking, Diabetes mellitus, Mortality, Coronary heart disease

## Abstract

**Background:**

As type 2 diabetes (T2D) patients have a high risk for coronary heart disease (CHD) and all-cause mortality and smoking is a major single risk factor for total and CHD mortality, it is important to understand the impact of smoking to the outcome events in comparison to people without T2D. Studies of excess risk of CHD incidence and mortality, and all-cause mortality in T2D patients related to smoking are controversial. We aimed to assess the risk of CHD incidence and mortality, and all-cause mortality in a large Finnish population cohort consisting of people with and without T2Daccording to smoking status.

**Methods:**

Prospective follow-up of 28 712 men and 30 700 women aged 25–64 years living in eastern and south-western Finland. The data on mortality were obtained from the nationwide death register using the unique national personal identification number. Follow-up information regarding CHD was based on the Finnish Hospital Discharge Register for non-fatal outcomes. The Cox proportional hazards models were used to estimate the association between diabetes and smoking subgroups and the risk for total and CHD mortality.

**Results:**

T2D patients who were smoking had higher all-cause mortality in both men (HR 3.76; 95% CI 2.95-4.78) and women (HR 4.51; 95% CI 2.91-7.00) than non-smoking diabetic men (HR 2.03; 95% CI 1.51-2.74) and women (HR 2.11; 95% CI 1.71-2.59). The CHD mortality risk for smoking men with T2D was higher (HR 6.15; 95% CI 4.22-8.96) than in non-smoking diabetic men (HR 2.62; 95% CI 1.60-4.29). Similar results were found in women revealing corresponding HR for CHD mortality of 6.92 (95% CI 2.79-17.19) for smoking, T2D women and 4.06 (95% CI 2.83-5.82) for non-smoking T2D women, respectively. Even though the risk of CHD incidence in T2D patients who had stopped smoking was statistically significantly higher than in their non-smoking non-diabetic counterparts, their CHD incidence was lower than in smoking T2D patients (HR in men 3.00; HR in women 2.80).

**Conclusion:**

It is important to address tobacco consumption in T2D patients, especially during primary health care contacts in order to reduce their high risk of CHD and all-cause mortality.

## Background

Type 2 diabetes (T2D) is one of the fastest growing public health problems in both developed and developing countries imposing a high financial burden on health care costs. The IDF has estimated that the numbers of adults with diabetes is expected to rise from 415 million in 2015 to 642 million by 2040 [1]. Most people with diabetes will sooner or later develop cardiovascular diseases (CVD) [2]. People with T2D have a higher risk of CVD and all-cause mortality than the rest of the population and once developing CVD their prognosis are poorer [3, 4]. Premature mortality caused by diabetes results in an estimated 12–14 years of life lost [5].

Studies of excess risk of coronary heart disease (CHD) and all-cause mortality in T2D patients related to smoking are controversial. This is surprising as smoking is one of the classical risk factors for CVD besides hypertension, hyperlipidaemia and diabetes, and contributes substantially to the global burden of disease [6, 7]. While some studies observed an additive increased risk of CHD and all-cause mortality [8–12], others did not find that smoking amplifies the risk of CHD or total mortality rates among T2D patients [13–17]. It has been argued that these conflicts may be attributed to low power or inadequate adjustment for confounders [18].

As T2D patients have shown to have a high risk for CHD and all-cause mortality and smoking is a major single risk factor for total and CHD mortality, it is important to understand the impact of smoking to these outcome events in people with T2D in comparison with people without T2D.

The aim of this study was to assess the risks of CHD and all-cause mortality in a large Finnish population cohort consisting of people with and without diabetes according to smoking status.

## Methods

### Study population

Five independent cross-sectional surveys were carried out at five-year intervals within the framework of the FINMONICA/FINRISK studies [19] between 1982 and 2002. An independent random sample was drawn from the national population register for each survey. The samples were stratified by sex and 10-year age categories according to the WHO MONICA protocol so that at least 250 people of each sex and 10-year age group were chosen [20]. The study participants included in the five surveys were 25 to 64 years of age, and the 1997 and 2002 surveys also included subjects aged 65 to 74 years. The participation rate varied in the survey cohorts between 63% and 83% in men and between 72% and 88% in women. The final sample comprised 28 712 men and 30 700 women.

### Baseline data collection

A self-administered questionnaire was mailed to the participants in advance. The questionnaire included questions on health behavior, such as smoking habits, and physical activity, education and medical history. Smoking habits were classified to three categories: never smokers, ex-smokers (those, who had smoked regularly but had stopped smoking at least six month before the survey) and current smokers.

The participants reported their occupational and leisure time physical activity. These were merged and regrouped into three categories (low, moderate and high) of total physical activity as described in previous publications [21, 22]. Education level, measured as the total number of school years, was divided into birth cohort-specific tertiles.

At the study site, specially trained nurses measured height, weight, and blood pressure (BP) using a standardized protocol [20]. Height and weight were measured without shoes and with light clothing. BP was measured twice from the right arm of the participant in sitting position after at least 5 min rest. A standard mercury sphygmomanometer was used. Starting from 1982, BP was measured twice and the mean of these two BP measurements was used in the analyses.

Serum total cholesterol was determined by using an enzymatic method (CHOD-PAP, Boehringer Mannheim, Mannheim, Germany). All samples were analyzed in the biochemical laboratory of the National Public Health Institute (nowadays National Institute for Health and Welfare), which is an accredited laboratory.

Participants who reported having diabetes on the questionnaire, or who had had a hospital discharge diagnosis of diabetes or the approval for free-of-charge medication for diabetes before the baseline survey were classified as having diabetes. Data on diabetes medication were ascertained from the national Social Insurance Institution’s register on special reimbursement for anti-diabetic drugs from 1964. Anti-diabetic drugs prescribed by a physician are reimbursed by social insurance in Finland and are subject to approval of a physician of the Institution who reviews each case history.

### CHD incidence and mortality

The original survey data were complemented by linkage to the nationwide death register of Statistics Finland according the unique national personal identification number that every Finnish resident has. These records covered the period from January 1970 to December 2008. The Eighth, Ninth and Tenth Revisions of the International Classification of Diseases (ICD) were used for coding the causes of death. The end point of the follow-up was the date of death.

Follow-up information regarding CHD was based on the Finnish Hospital Discharge Register for non-fatal outcomes. The ICD codes used for CHD incidence and mortality were as follows: non-fatal myocardial infarction 410–411 (ICD9) and I21–I22 (ICD10) and fatal cases of CHD 410–414 (ICD9) and I20–I25 (ICD10). The end points during follow-up was incident CHD, defined as either the first non-fatal CHD or CHD death. The calendar period of follow up was truncated by the same date, 31/12/2008, as death information.

### Statistical analysis

Statistical analyses were performed using SAS 9.2. The Cox proportional hazards models were used to estimate the association between diabetes and smoking subgroups and the risk for total and CHD mortality. Analyses were adjusted first for age, study area and study year (A) then for age, study region, study year, education, systolic blood pressure (SBP), cholesterol level, physical activity, alcohol consumption and body mass index (BMI) (B). The data of all six surveys were pooled together as no first level interaction were found between diabetes/smoking groups and these variables regarding CHD, stroke or total mortality, respectively incidence. The proportional hazards assumption in the Cox model was tested based on the Schoenfeld Residuals. Estimated hazard ratios (HR) and their 95% confidence intervals (CI) are presented.

## Results

The baseline characteristics of the study participants are presented in Table 1 according to sex and smoking status. Among men with T2D 32% were current and 37% ex-smokers. The corresponding prevalence of smoking and ex-smoking in men without T2D was 40% and 26%, respectively. Among women with T2D 13% were current and 11% ex-smokers. The corresponding prevalence of smoking and ex-smoking in women without T2D was 17% and 10%, respectively. People with T2D seemed to have a higher prevalence of overweight and obesity, lower levels of physical activity, higher serum cholesterol and systolic blood pressure level than people without diabetes at baseline or during follow-up. This pattern was observed in both men and women.

**Table 1:** Baseline characteristics of the study population according to diabetes and smoking status

	No diabetes			Diabetes		
	Non-smokers	Ex-smokers^a^	Smokers^b^	Non-smokers	Ex-smokers	Smokers
Men (n)	9539	7063	11054	323	392	341
Age (years)	43.2 (11.9)^c^	48.5 (11.7)	42.6 (11.1)	54.1 (11.5)	56.9 (9.1)	51.0 (10.4)
Education (%)						
Low	28	29	37	31	31	33
Moderate	33	37	35	31	35	34
High	39	34	28	38	34	33
Body mass index (kg/m^2^)	26.1 (3.5)	27.3 (3.7)	25.8 (3.7)	28.5 (4.4)	29.3 (4.0)	27.8 (4.9)
Systolic blood pressure (mmHg)	140 (18)	144 (20)	142 (19)	148 (24)	148 (21)	146 (21)
Serum cholesterol (mmol/l)	5.8 (1.2)	6.1 (1.2)	6.2 (1.31)	5.7 (1.2)	5.9 (1.3)	6.1 (1.4)
Total physical activity (%)						
Low	8	10	13	16	21	24
Moderate	83	84	82	81	77	75
High	9	6	5	3	2	1
Women (n)	21463	2980	5101	878	125	153
Age (years)	45.7 (11.6)	42.5 (11.6)	40.4 (10.8)	54.0 (11.1)	51.2 (11.9)	46.7 (11.0)
Education (%)						
Low	32	31	40	36	31	38
Moderate	34	33	33	34	39	32
High	34	36	27	30	30	30
Body mass index (kg/m^2^)	26.2 (4.8)	25.9 (4.6)	24.9 (4.5)	29.7 (6.1)	31.1 (6.3)	28.4 (5.4)
Systolic blood pressure (mmHg)	140 (23)	131 (19)	131 (19)	148 (24)	145 (23)	138 (23)
Serum cholesterol (mmol/l)	6.0 (1.3)	5.5 (1.2)	5.6 (1.2)	6.0 (1.3)	5.7 (1.1)	6.0 (1.3)
Total physical activity (%)						
Low	15	13	17	27	29	28
Moderate	82	83	80	71	70	70
High	3	4	3	2	1	2

^a^those, who had smoked regularly but had stopped smoking at least six month before the survey

^b^those who currently smoke or those who had stopped smoking less than six months before the survey

^c^mean and standard deviation

### T2D, smoking and all-cause mortality

The HRs for all-cause mortality were higher for T2D patients compared with men and women without T2D. T2D patients who were smoking had higher all-cause mortality in both men (HR 3.76; 95% CI 2.95-4.78) and women (HR 4.51; 95% CI 2.91-7.00) than non-smoking diabetic men (HR 2.03; 95% CI 1.51-2.74) and women (HR 2.11; 95% CI 1.71-2.59) (Table 2). T2D patients that had stopped smoking also had increased all-cause mortality compared with non-smoking, diabetic men and women when adjusted for age, study area and study year, education, systolic blood pressure, physical activity, serum cholesterol and BMI (men: HR 2.56; 95% CI 2.04-3.22), women: HR 2.61; 95% CI 1.57-4.31).

**Table 2:** Hazard Ratio (HR) of all-cause mortality among men and women with and without diabetes at baseline according to smoking status

	Model A^b^			Model B^c^	
	Mortality rate^a^	HR	95% CI	HR	95% CI
Men					
Non-diabetic, non-smokers	0.88	1	Ref^d^	1	Ref
Non-diabetic, ex-smokers	1.52	1.46	1.29-1.66	1.38	1.21-1.57
Non-diabetic, smokers	1.93	2.98	2.65-3.35	2.63	2.33-2.97
Diabetic, non-smokers	2.63	2.22	1.66-2.95	2.03	1.51-2.74
Diabetic, ex-smokers	3.30	2.94	2.35-3.67	2.56	2.04-3.22
Diabetic, smokers	3.97	4.35	3.44-5.51	3.76	2.95-4.78
Women					
Non-diabetic, non-smokers	0.92	1	Ref	1	Ref
Non-diabetic, ex-smokers	0.44	1.27	1.02-1.59	1.30	1.04-1.63
Non-diabetic, smokers	0.85	2.41	2.06-2.81	2.37	2.02-2.78
Diabetic, non-smokers	2.43	2.40	1.96-2.94	2.11	1.71-2.59
Diabetic, ex-smokers	1.75	3.25	1.98-5.36	2.61	1.57-4.31
Diabetic, smokers	1.71	4.96	3.21-7.67	4.51	2.91-7.00

^a^number of events per 100 person-years

^b^adjusted for age, study area and study year

^c^adjusted for age, study area, study year, education, systolic blood pressure, physical activity, serum cholesterol and BMI

^d^reference group

### T2D, smoking and CHD mortality

Table 3 shows the hazard ratios for CHD mortality among men and women with and without diabetes according to smoking status. The CHD mortality risk for smoking men with T2D was higher (HR 6.15; 95% CI 4.22-8.96) than in non-smoking diabetic men (HR 2.62; 95% CI 1.60-4.29). Similar results were found in women revealing corresponding HR for CHD mortality of 6.92 (95% CI 2.79-17.19) for smoking, T2D women and 4.06 (95% CI 2.83-5.82) for non-smoking T2D women, respectively. T2D patients that had stopped smoking also had a higher risk of CHD mortality compared with non-smoking, diabetic men and women when adjusted for age, study area and study year, education, systolic blood pressure, physical activity, serum cholesterol and BMI (men: HR 4.30; 95% CI 3.04-6.08), women: HR 5.00; 95% CI 2.15-11.64). The HRs of CHD mortality in smoke-free T2D patients was statistically significantly higher compared with non-smoking people without T2D (men: HR 2.62; 95% CI 1.60-4.29; women: HR 4.06; 95% CI 2.83-5.82).

**Table 3:** Hazard Ratio (HR) of coronary heart disease (CHD) mortality among men and women with and without diabetes at baseline according to smoking status

	Model A^b^			Model B^c^	
	Mortality rate^a^	HR	95% CI	HR	95% CI
Men					
Non-diabetic, non-smokers	0.29	1	Ref^d^	1	Ref
Non-diabetic, ex-smokers	0.56	1.71	1.36-2.16	1.59	1.25-2.02
Non-diabetic, smokers	0.65	3.29	2.64-4.10	3.11	2.48-3.92
Diabetic, non-smokers	1.06	2.86	1.77-4.61	2.62	1.60-4.29
Diabetic, ex-smokers	1.63	5.28	3.77-7.40	4.30	3.04-6.08
Diabetic, smokers	1.73	6.98	4.85-10.03	6.15	4.22-8.96
Women					
Non-diabetic, non-smokers	0.25	1	Ref	1	Ref
Non-diabetic, ex-smokers	0.08	1.33	0.74-2.37	1.40	0.78-2.51
Non-diabetic, smokers	0.20	3.43	2.40-4.89	3.84	2.65-5.55
Diabetic, non-smokers	0.96	4.81	3.37-6.85	4.06	2.83-5.82
Diabetic, ex-smokers	0.75	7.73	3.37-17.73	5.00	2.15-11.64
Diabetic, smokers	0.57	8.79	3.56-21.67	6.92	2.79-17.19

^a^number of events per 100 person-years

^b^adjusted for age, study area and study year

^c^adjusted for age, study area, study year, education, systolic blood pressure, physical activity, serum cholesterol and BMI

^d^reference group

### T2D, smoking and CHD incidence

Smoking T2D patients had an increase in CHD incidence compared with non-smoking people with and without T2D (Table 4). The risk increase in smoking diabetic men was 3.27-fold (95% CI 2.45-2.40) and 4.55-fold in women (95% CI 2.48-8.33) when controlled for age, education, systolic blood pressure, physical activity, serum cholesterol and BMI. Even though the risk of CHD incidence in T2D patients who had stopped smoking was statistically significantly higher than in their non-smoking non-diabetic counterparts, the CHD incidence was lower than in smoking T2D patients (HR in men 3.00; HR in women 2.80).

**Table 4:** Hazard Ratio (HR) of coronary heart disease incidence among men and women with and without diabetes at baseline according to smoking status

	Model A^b^			Model B^c^	
	Incidence rate^a^	HR	95% CI	HR	95% CI
Men					
Non-diabetic, non-smokers	0.69	1	Ref^d^	1	Ref
Non-diabetic, ex-smokers	1.10	1.25	1.08-1.44	1.15	0.99-1.32
Non-diabetic, smokers	1.21	2.04	1.79-2.33	1.95	1.70-2.23
Diabetic, non-smokers	1.85	1.70	1.19-2.44	1.56	1.08-2.24
Diabetic, ex-smokers	2.98	3.66	2.87-4.68	3.00	2.33-3.85
Diabetic, smokers	2.69	3.51	2.64-4.67	3.27	2.45-4.36
Women					
Non-diabetic, non-smokers	0.50	1	Ref	1	Ref
Non-diabetic, ex-smokers	0.19	1.21	0.88-1.66	1.27	0.92-1.74
Non-diabetic, smokers	0.39	2.13	1.70-2.67	2.32	1.84-2.92
Diabetic, non-smokers	1.52	2.94	2.30-3.77	2.60	2.02-3.35
Diabetic, ex-smokers	0.99	3.59	1.91-6.76	2.80	1.48-5.30
Diabetic, smokers	1.24	5.37	3.02-9.58	4.55	2.48-8.33

^a^number of events per 100 person-years

^b^adjusted for age, study area and study year

^c^adjusted for age, study area, study year, education, systolic blood pressure, physical activity, serum cholesterol and BMI

^d^reference group

## Discussion

This study showed that smoking men and women with T2D had a higher risk of CHD incidence, and CHD and all-cause mortality compared with non-smoking people free of diabetes. Stopping smoking reduced the risk of all-cause mortality in both men and women with T2D. Whereas T2D patients who were not smoking or who had stopped smoking had an increased risk of CHD incidence and mortality, that increase seemed to be lower than the one observed in their smoking diabetic counterparts.

As smoking may influence various mechanisms proposed to increase the risk of CHD (e.g. vascular endothelial injury, increased oxidative stress, thromboembolism), it is possible that the effect of smoking on CHD and MI might be more dramatically increased in diabetic patients, by a combination of short-term effects (coronary artery spasm, arrhythmias) and long-term effects (metabolic and thrombotic disorders) on the cardiovascular system [23, 24]. In T2D patients, smoking is an independent risk factor for CHD, stroke and peripheral vascular disease [8, 25–27]. It accentuates the dyslipidemia of T2D that is associated with increased hepatic lipase activity that may produce atherogenic, small, dense LDL particles [28].

In agreement with our study, the Nurses’ Health Study showed that the age-adjusted incidence rate of total CHD was much higher among diabetic women than among non-diabetic women of similar smoking status. However, they observed a considerably higher hazard ratio for CHD among diabetic women than for non-diabetic women of similar smoking status compared with our study [8] presenting a multivariate adjusted HR 7.7 for CHD in women with T2D smoking more than 15 cigarettes/day compared with non-diabetic, nonsmoking women [8]. Furthermore, current smokers seem to be at higher risk than ex-smokers in regard all-cause and CHD mortality. These findings are in agreement with previous studies showing that previous smokers might still suffer from the adverse effects related to smoking even though the risk started to decrease [8–10].

Most studies have compared mortality rates according to smoking status among patients with T2D rather than comparing mortality rates with the general population [7, 9–11]. A cohort study involving 13 087 female and male patients with T2D from the Swedish National Diabetes Register revealed statistically significant hazard ratios for smoking and first-incident fatal/nonfatal MI and total mortality of 1.7 and 1.8, respectively [7]. The Nurses’ Health Study reported that cigarette smoking was associated in a dose–response manner with an increased mortality among women with type 2 diabetes [9] and the United Kingdom prospective diabetes study found that hazard ratio for smokers in regard CHD of 1.41 (1.06 to 1.88) among a 7.9 year follow-up of 3055 T2D patients (10). Finally, a three year follow-up of 390 elderly T2D patients (mean age 73 years) showed an increased the risk of mortality 2.58 (95% CI 1.30–5.11) among the smoking T2D patients compared with the non-smoking ones [11].

Interestingly, some cohort studies did not find that smoking increased the risk of CHD or all-cause mortality among T2D patients [12–17]. The Aerobics Center Longitudinal Study included 2316 men with T2D which were followed-up for almost 16 years [12] and the Rochester Epidemiology Project comprised 337 T2D patients of Olmsted County, Minnesota [17]. Even though both studies reported increased risks for current and ex-smoking in regard CVD or all-cause mortality, this risk increase did not reach statistical significance. Another prospective population-based study of 400 patients with T2D in Skara, Sweden revealed a 1.66-fold risk increase for all-cause mortality among smokers compared with non-smokers with a 95% CI of 0.99-2.76 [14]. Probably, the latter two studies may have a low power to detect risk increases due to a small sample size [14, 17]. Furthermore, both the ZODIAC-10 study and the Milan Study on Atherosclerosis and Diabetes were lacking adequate adjustments of their results [15, 16, 18].

A recent meta-analysis of observational prospective studies assessing the excess risk of mortality and CVD events associated with smoking among patients with diabetes revealed a relative risk comparing smokers with nonsmokers of 1.48 for total mortality 1.54 for CHD [18]. Furthermore, that excess risk was observed among former and current smokers with a greater risk in current smokers.

The life-extending effect of successful smoking cessation ultimately concerns every smoking patient in medical practice and physicians should have an ethical obligation to educate their patients about smoking and should not hesitate to routinely advise to quit [29, 30]. In Finland, unfortunately, smoking is not discussed during most routine health care consultations with smokers if they do not present any “smoking-related” disease [31]. Physicians should ask their patients whether they smoke, how much they smoke, and whether they are willing to quit. As a subsequent step one should explain to every smoking patient why and to what extent smoking cessation would, for medical reasons, favorably change their future health. This short talk hardly takes more than two minutes [29]. It has been shown that if the patient presents CHD, approximately eight out of ten Finnish primary care physicians give smoking cessation advice to that specific population group [31]. However, smoking counseling should be given long before the patients have CHD and especially if they have T2D as pointed out in the current clinical guidelines for diabetes care [32].

Naturally, our study had some limitations. The baseline assessment of our cohort is limited to the examination on a single day when participants entered the study, as typical for large cohort studies. It cannot account for changes in lifestyle or risk factors of CHD disease leading to a shift of participants between categories during the study period. Furthermore, we cannot completely exclude the effects of confounding due to some unmeasured dietary and other lifestyle factors that may influence CHD or all-cause mortality. In addition, assessing smoking habits by questionnaire may lack some of accuracy as we did not have information regarding pack-years or intensity of smoking. However, self-reported smoking habits are commonly used in epidemiological studies and seem to be rather accurate when compared with biochemical markers of tobacco use.

## Conclusions

It is important to address tobacco consumption in T2D patients in primary health-care in order to reduce their risk of CHD and all-cause mortality. Smoking cessation programs should be offered to each T2D patient and former smoking T2D patients should be encouraged to remain smoke-free. As smoking and ex-smoking T2D patients have a higher risk of CHD incidence and mortality, their health status with regard to CHD should be carefully checked during their annual follow-up control visits with health care personnel.
